# Supplementary material for: Evaluation and Discrimination of Lipid Components and Iron and Zinc Levels in Chicken and Quail Eggs Available on the Polish Market
Source: Foods. 2024 May 17;13(10):1571. doi: 10.3390/foods13101571 (PMC11121015; doi:10.3390/foods13101571)
Supplement: Supplementary file 1 [file foods-13-01571-s001.zip › foods-2982878-supplementary.pdf]

Table S1 Correlation coefficients between lipid component content and iron in chicken and quail egg yolk

	General	E	F	B	C	N3	GL	Q
C14:0	-0.57	0.01	-0.01	0.35	0.27	0.30	-0.31	0.19
C14:1	-0.50	-0.18	-0.20	0.36	0.17	0.33	-0.28	0.50
C15:0	-0.65	-0.20	0.21	0.01	0.23	0.19	0.04	0.22
C16:0	-0.56	-0.09	-0.18	0.28	0.20	0.22	-0.19	0.06
C16:1	-0.54	-0.21	-0.27	0.31	0.25	0.29	-0.35	0.35
C17:0	-0.51	0.03	0.32	0.07	-0.04	0.04	0.11	-0.01
C17:1	-0.63	-0.04	-0.06	0.04	0.11	-0.07	0.12	0.14
C18:0	-0.45	-0.06	0.07	0.32	0.08	0.18	-0.08	-0.14
c9 C18:1 OL	-0.58	-0.01	-0.33	-0.06	0.08	0.08	-0.28	-0.10
c11 C18:1	-0.63	-0.21	-0.19	0.08	0.02	0.05	-0.20	0.30
C18:2 n6 LA	-0.46	0.02	0.16	0.37	-0.05	0.15	-0.09	0.33
C18:3 n6 GLA	-0.33	-0.05	0.42	0.06	0.33	0.23	0.16	0.36
C18:3 n3 ALA	-0.12	0.03	-0.01	0.44	0.14	-0.36	-0.05	-0.15
C20:0	-0.56	-0.28	-0.05	0.37	0.26	0.31	-0.25	0.06
C20:1	-0.59	-0.18	-0.12	0.30	0.13	0.16	-0.30	-0.08
C21:0	-0.42	0.02	0.20	0.55	0.08	0.23	-0.19	0.24
C20:2	-0.09	-0.50	0.11	-0.05	0.08	0.15	-0.19	0.23
C20:3 n6	-0.71	-0.32	0.31	0.18	0.29	0.30	-0.27	0.27
C20:4 n6 AA	-0.58	-0.15	0.40	-0.04	0.11	0.24	0.27	-0.05
C22:0	-0.34	0.01	-0.45	0.29	0.50	0.05	-0.20	0.11
C20:5 EPA	0.12	-0.65	0.02	-0.08	-0.02	-0.37	0.08	-0.22
C22:2	-0.58	-0.14	-0.15	-0.39	0.02	-0.08	-0.26	-0.18
C24:0	0.15	-0.16	-0.03	-0.11	0.22	0.60	0.00	0.47
C22:5	0.07	-0.04	-0.09	0.16	0.50	-0.36	0.21	-0.27
C22:6 n3 DHA	-0.33	-0.10	-0.12	0.30	0.35	-0.47	0.43	-0.32
Cholesterol	-0.05	0.09	0.39	-0.42	-0.05	0.00	-0.04	-0.16

The red colour of the font indicates $p < 0.05$

E - chicken eggs from organic production, F - free range chicken eggs, B - barn chicken eggs, C - chicken eggs from caged hens, N3 - chicken eggs with an increased content of n3 fatty acids, GL - chicken eggs from Green-legged Partridge, Q - partridge quail eggs

Table S2. Correlation coefficients between lipid component content and zinc in in chicken and quail egg yolk

	General	E	F	B	C	N3	GL	Q
C14:0	-0.14	0.15	-0.22	-0.08	0.02	0.34	-0.14	-0.05
C14:1	-0.03	0.16	-0.31	-0.12	-0.03	0.61	0.01	-0.45
C15:0	-0.12	-0.04	-0.05	0.22	-0.01	0.13	-0.09	-0.76
C16:0	-0.28	-0.06	-0.38	-0.29	-0.04	0.21	-0.05	-0.23
C16:1	-0.11	0.14	-0.33	-0.13	0.00	0.46	-0.11	-0.30
C17:0	-0.31	-0.24	-0.05	-0.25	0.06	-0.12	-0.27	0.05
C17:1	-0.12	-0.01	-0.18	-0.19	-0.06	-0.27	-0.25	-0.28
C18:0	-0.40	-0.37	-0.19	-0.12	0.01	0.16	-0.08	-0.23
c9 C18:1 OL	-0.34	-0.07	-0.35	-0.23	-0.04	-0.17	-0.05	-0.07
c11 C18:1	-0.06	0.07	-0.21	-0.10	-0.06	0.09	-0.14	0.10
C18:2 n6 LA	-0.11	-0.10	0.02	-0.10	0.07	0.04	-0.07	0.04
C18:3 n6 GLA	-0.40	-0.24	0.03	-0.17	0.13	0.16	-0.25	-0.18
C18:3 n3 ALA	-0.10	-0.26	-0.08	0.12	-0.17	-0.63	-0.14	0.45
C20:0	-0.09	-0.09	-0.20	-0.17	-0.09	0.32	-0.15	-0.26
C20:1	-0.01	-0.13	-0.07	0.14	-0.06	-0.08	-0.01	-0.34
C21:0	0.25	0.00	0.05	0.05	0.14	0.19	-0.05	-0.55
C20:2	0.23	0.21	0.07	0.06	0.04	0.49	-0.07	-0.39
C20:3 n6	-0.07	-0.07	0.05	-0.20	0.11	0.06	0.02	-0.52
C20:4 n6 AA	-0.21	-0.19	-0.06	-0.21	0.09	0.21	-0.13	-0.20
C22:0	-0.27	-0.22	-0.47	-0.11	0.20	-0.15	0.37	-0.07
C20:5 EPA	-0.22	0.23	-0.19	-0.24	-0.59	-0.48	0.07	0.33
C22:2	-0.07	0.01	-0.06	0.00	0.10	-0.31	0.04	-0.17
C24:0	-0.36	-0.30	0.02	-0.23	0.31	0.60	-0.18	-0.34
C22:5	-0.66	-0.55	-0.22	-0.10	-0.05	-0.65	-0.30	0.28
C22:6 n3 DHA	-0.41	-0.40	-0.25	-0.04	-0.14	-0.79	-0.07	0.73
Cholesterol	-0.37	0.02	0.18	-0.26	-0.34	-0.03	0.29	-0.46

The red colour of the font indicates $p < 0.05$

E - chicken eggs from organic production, F - free range chicken eggs, B - barn chicken eggs, C - chicken eggs from caged hens, N3 - chicken eggs with an increased content of n3 fatty acids, GL - chicken eggs from Green-legged Partridge, Q - partridge quail eggs
